# A Review of the Literature of Surgical and Nonsurgical Treatments of Invasive Squamous Cells Carcinoma

**DOI:** 10.1155/2018/9489163

**Published:** 2018-04-02

**Authors:** Concetta Potenza, Nicoletta Bernardini, Veronica Balduzzi, Luigi Losco, Alessandra Mambrin, Anna Marchesiello, Ersilia Tolino, Sara Zuber, Nevena Skroza, Ilaria Proietti

**Affiliations:** ^1^Dermatology Unit “Daniele Innocenzi”, Department of Medical-Surgical Sciences and Biotechnologies, Sapienza University of Rome, Polo Pontino, Rome, Italy; ^2^Plastic Surgery Unit, Department of Surgery, Sapienza University of Rome, Rome, Italy

## Abstract

Cutaneous squamous cell carcinoma (cSCC) is an increasing public health problem. It is a primary malignant skin tumor with Malpighian differentiation and together with basal cell carcinoma is classified among nonmelanoma skin cancers (NMSCs). cSCC usually occurs on photoexposed areas, such as the head, the neck, and the extremities, and its incidence increases with age. Invasive forms of this skin tumor tend to be more aggressive showing a higher metastatic potential, usually regarding regional lymph nodes. Treatment options for invasive cSCCs include both surgical and nonsurgical options. The therapeutic choice depends on several factors, such as anatomic location, risk factors for tumor recurrence, age, and health status of the patient. This review aims to provide an overview of the current evidence on therapeutic surgical and nonsurgical management of invasive cSCC.

## 1. Introduction

Cutaneous squamous cell carcinoma (cSCC) is the second most common form of NMSC after basal cell carcinoma. In 2017, the American Joint Committee on Cancer revised the staging guidelines of this tumor reflecting the recent evidence on high risk clinicopathologic features [1].

The most significant modification from the prior seventh edition is the introduction of cSCC from a general chapter for the entire body to a head and neck-specific cutaneous malignancies chapter, thus addressing NMSCs of the head and neck as well as those malignancies that arise from the mucosal surfaces of the upper aerodigestive tract and salivary glands.

This skin tumor, in fact, is characterized by the malignant proliferation of keratinising cells which mimics those of the spinous layer of the epidermis [2–4]. These cells can migrate beyond the level of the dermoepidermal junction, in the dermis or deeper, and may develop both de novo and from precursor lesions, such as AK and Bowen's diseases, thus becoming invasive forms. These forms are aggressive and express a higher metastatic potential, usually regarding regional lymph nodes.

**Figure 1:**
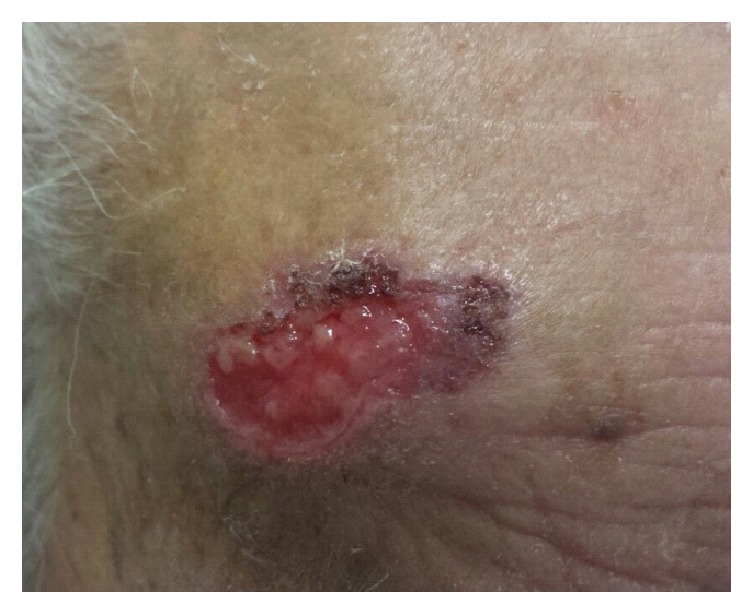
Squamous cell carcinoma presenting on forehead in the form of enlarging ulcer.

**Figure 2:**
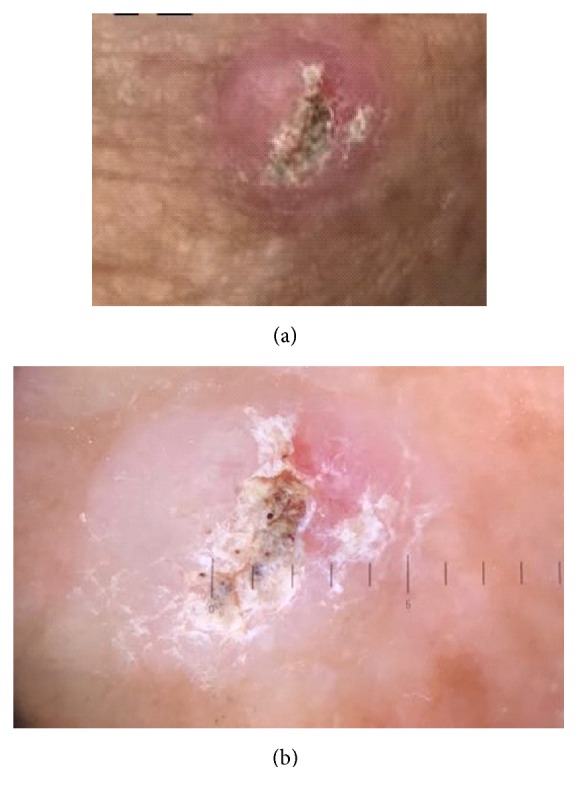
Clinical (a) and dermoscopic (b) aspects of a firm, erythematous hyperkeratotic Squamous Cell Carcinoma.

**Figure 3:**
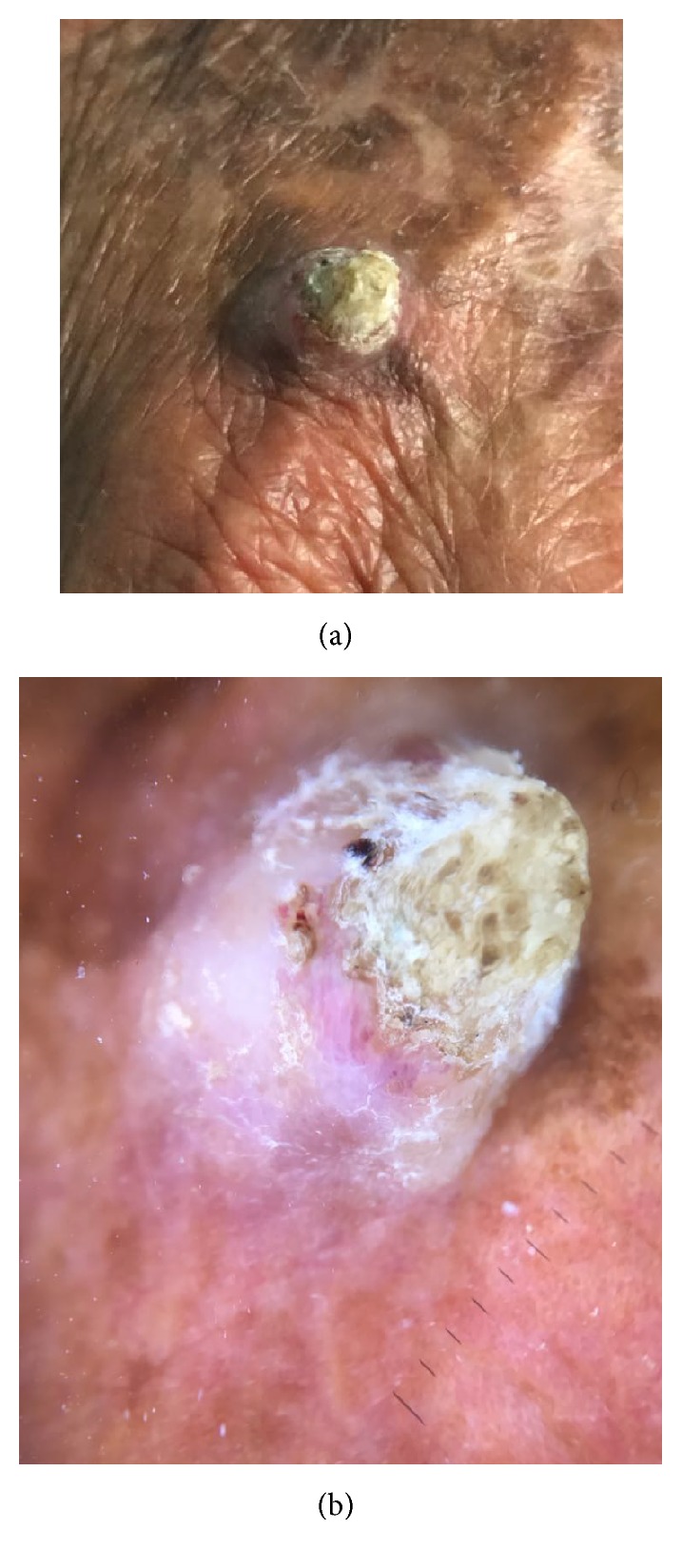
Clinical (a) and dermoscopic (b) images of cutaneous horn.

Clinically, it usually presents as a firm, flesh colored or erythematous, hyperkeratotic enlarging plaque or papule, but it may also be pigmented or verrucous (Figures 1, 2(a) and 2(b)). Sometimes cSCC may appear as an ulcer, a smooth nodule or a tricky cutaneous horn (Figures 3(a) and 3(b)).

cSCC arises frequently in field of cancerization rather than de novo. Genetic alterations are recognizable both in tumoral cells and in elements without histological atypia, so microenvironment of premalignant lesions can influence their progression to invasive and metastatic cSCC [5, 6]. The genetic background also influences malignant potential of cSCC, as Genome-wide association studies (GWAS) have identified genetic loci associated with cSCC risk, and single nucleotide polymorphisms (SNP) of the class II human leukocyte antigen region associations with tumor development have recently been investigated [6].

cSCC results from the malignant proliferation of epidermal keratinocytes due to complex biological events involving multiple factors [5, 6]. Ultraviolet radiations (UVs), particularly UVB (290–320 nm) and UVA (320–340 nm) radiations, have a pivotal role in tumor pathogenesis. Sun exposure and artificial sources of UV, like PUVA therapy, are major epidemiologic risk factors for cSCC [7]. Genetic disorders such as xeroderma pigmentosum and chronic skin damaged areas including scars, ulcers, burn sites, and chronic sun exposure are related to increased incidence of skin cancer spread. Recent evidence displayed that immunosuppressed patients tend to develop multiple and more aggressive cSCC; precisely patients undergoing solid organ transplantation have 65-fold higher risk of developing cSCC than the general population [5, 7, 8]. It is also important to evaluate the location of the primary skin lesions.

Tumors located on lips and ears are associated with higher rates of local recurrence and distant metastasis (20–50%); in these cases lateral-cervical, submandibular, submental, and intraparotid lymph nodes are frequently involved [6].

Tumor thickness is currently considered to be the most important independent predictor of metastasis in cSCC. Perineural invasion increases the risk of recurrence, according to the thickness of the nerves affected and to the presence of clinical and/or radiologic signs of invasion. 0.1 mm is the cutoff for nerve diameter associated with poor short-term and long-term prognosis.

cSCC has also been described in melanoma patient undergoing BRAF inhibitors (BRAFi) treatment. These drugs induce therapeutic responses in metastatic melanoma but also develop secondary malignant skin tumors. This is explained in part by “paradoxical ERK activation,” or the hyperactivation of ERK signaling by BRAF inhibitor in BRAF wild-type cells [9, 10].

A trend of increasing incidence for cSCC, since the 1960s, has been registered [11], probably related to the ageing of the population and to the change of sun exposure behaviour; in fact, 80% of people affected are older than 60 years. It is estimated that over 700.000 new cases of cSCC are diagnosed annually in the USA [3]; moreover the cSCC incidence has been predicted to increase more than 50% by 2030 [12, 13].

## 2. Treatment Options

Treatment options for invasive SCC include both surgical and nonsurgical modalities. The therapeutic choice depends on several factors, such as anatomic location, risk factors for tumor recurrence, age, and health status of the patient [14]. The TNM classification, developed by AJCC/IUAC/UICC, which is used for all skin cancers except melanoma, is not suitable for SCC, since it is not considered in the multiple prognostic criteria identified in the literature.

The principal aim of treatment is to completely remove or destroy the tumor, while preserving the function and the aesthetic appearance.

Surgery still represents the gold standard approach and may be combined with plastic reconstruction, Mohs' micrographic surgery (MMS), electrodessication, or curettage.

Nonsurgical options for invasive cSCC include topical* chemotherapy*, topical* immune response* modifiers,* radiotherapy*, and systemic chemotherapy. The latter is usually reserved for patients with metastatic lesions.

Such approaches are recommended only when the patient refuses surgery or surgery cannot be performed.

### 2.1. Surgery

**Figure 4:**
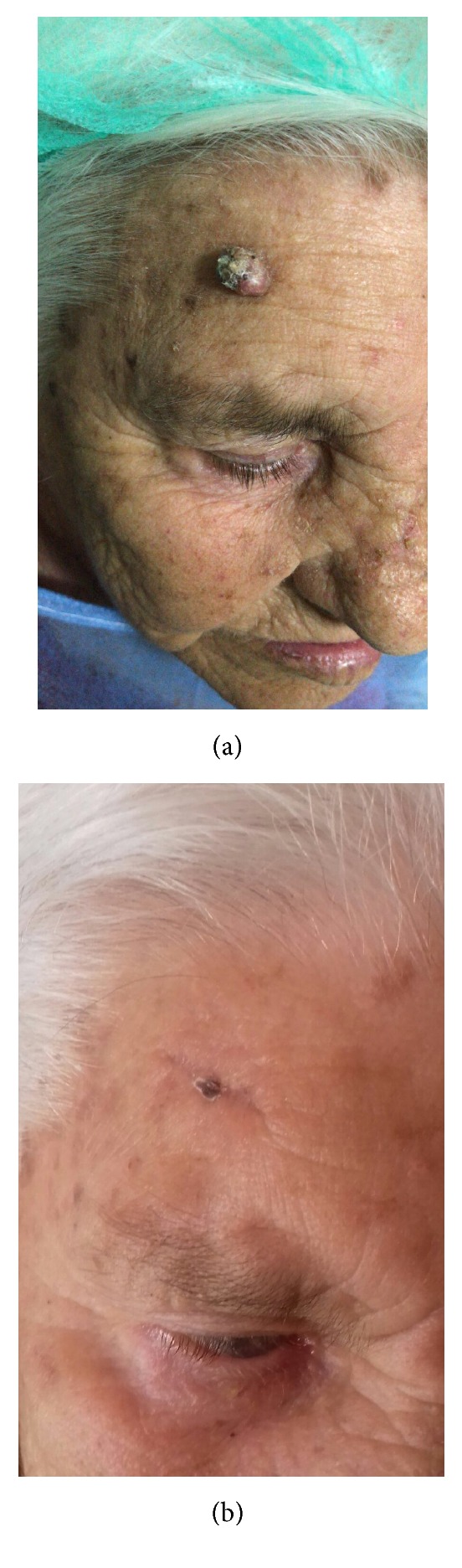
Before (a) and after (b) surgical excision of hyperkeratotic nodular squamous cell carcinoma.

Complete surgical removal with histopathological control of excision margins represents the first line treatment for cSCC (Figures 4(a) and 3(b)). The main aim of surgery is to obtain complete tumor excision while preserving the function and a satisfactory cosmetic result, particularly in sensitive areas such as lips, noise, ears, and natural orifices. Surgery is also indicated with adjuvant radiation to control regional disease in the presence of nodal metastases and perineural invasion. However predicting the risk of developing lymph node metastases from cSCC is difficult and clinical data about the utility of sentinel node biopsy (SNB) is lacking in literature. This leads physicians to be uncertain on which patients require staging and what procedure to use in nodal staging. So further studies are needed to define the role of SLNB in patients with cSCC.

As for the surgical treatment, two techniques can be performed: standard surgical excision with postoperative margin assessment or micrographic surgery and its variants: MMS and “slow Mohs” technique.

Standard excision allows confirming the histological diagnosis of cSCC and verifying the complete removal of the tumor. Excision margins refer to the minimum amount of healthy skin over the visible limits of the tumor that should be removed to assure the complete tumor eradication [15]. Excision margins should be adapted to the clinical size and degree of tumor aggression. A prospective study involving 141 cases of cSCC showed that a 4 mm margin allows for completely removal of 95% of low risk tumors, measuring less than 2 cm in diameter [16]. Larger tumors (>2 cm) require more than 6 mm excision margins. Moreover, in tumors with high risk prognostic factors, such as moderate or poor differentiation, recurrent tumor, perineural invasion, extension deep into the subcutaneous fat, and/or location on the ear or lip, more than 6 mm margins are recommended, independently of the clinical diameter [17].

### 2.2. Microscopically Controlled Surgery (Mohs' Micrographic Surgery)

Microscopically controlled surgery (MCS) or Mohs' micrographic surgery (MMS) was introduced in the first half of the 20th century as an alternative to standard excision, electrodessication, and radiation therapy for cutaneous carcinomas.

MMS consists of the removal of serial horizontal sections of the tumor margins, in order to minimize the risk of recurrence [18]. The main aim of MMS is to completely remove the tumor while sparing as many tissues as possible.

At first, the tumor is surgically removed with minimal margins; then thin horizontal sections (2 mm) of the surrounding skin are topographically marked and removed and histologically analyzed in an extemporaneous fashion. If the margins are positive for tumor cells, localized reexcisions are performed until the area is completely tumor free.

Tumor slices are examined intraoperatively using frozen sections (Mohs surgery) or on paraffin sections (“slow Mohs” surgery).

The disadvantages of MMS consist of a longer duration of the operation and higher costs.

MMS demonstrated a 5-year recurrence rate for cSCC compared to standard surgery of 3% versus 8%, respectively, and should be recommended in selected cases [19, 20]:

- In high risk cSCCs as first line treatment [21]
- In low risk cSCCs when the margins are positive after standard excision with 4–6 mm margins [21]
- When complete excision is difficult to achieve [22]
- In case of high risk of recurrence
- For cSCCs localized in sensitive sites such central facial region and periorificial areas, nose and lips [22]
- In cases where surgical excision could cause functional impairment [23]
- In tumors with aggressive histological growth patterns [24].

### 2.3. Radiotherapy

Radiotherapy is based on the administration of ionizing radiations for skin cancer treatment through two different techniques: external radiotherapy and interstitial curietherapy (brachytherapy).

External radiotherapy uses superficial or deep X-rays, gamma rays (telecobalt), or electron beams (linear accelerators) [7]. Different regimens have been used up to date, which vary in terms of duration, fractioning, and total dose administered [25]. The NCCN recommended an algorithm consisting of a total dose of 45–50 Gy in fractions of 2.5–3 Gy for SCCs <2 cm in diameter and 60–66 Gy in fractions of 2 Gy or 50–60 Gy in fractions of 2.5 Gy for tumors >2 cm [26].

Radiation field involves both the tumor and a safety margin of 1–1,5 cm of surrounding skin.

Radiotherapy is recommended for cSCC in the following cases:

- It is recommended as an alternative to surgery when patient refuses surgery or patient's conditions contraindicate surgery [27].
- It is recommended as a primary treatment for inoperable SCCs or in the adjuvant setting [27, 28].
- It is recommended for debilitated patients who cannot tolerate extensive surgery.
- It is recommended when surgical excision would be extremely disfiguring.
- In recurrent cSCCs, radiotherapy should be considered as adjuvant therapy to improve tumor control.
- It is recommended in management of metastasis.
- It is recommended when tissue margins are not tumor-free after surgical excision.
- Adjuvant radiotherapy should be recommended in all patients affected by aggressive SCCs with perineural invasion and for individuals who have undergone lymph node dissection with nodal disease of the head and neck region [26].
- It is recommended in case of SCCs involving problematic sites such as the face or hands.
- Finally, radiotherapy should be considered in immunosuppressed patients.

Radiotherapy is not recommended in verrucous SCCs [29], in patients with genodermatoses predisposing to skin cancers such as xeroderma pigmentosum or Gorlin-Goltz syndrome, and in patient with connective tissue disease (e.g., systemic sclerosis).

Radiotherapy is contraindicated on photodamaged skin and in previously irradiated areas, for cSCCs localized in poorly vascularized or traumatized sites and for advanced lesions invading bones, joints, or tendons [27].

Acute and chronic side effects (radiodermatitis) are commonly associated with radiotherapy administration, the latter including pigmentary changes, atrophy, hair loss, fibrosis, lymphedema, and telangiectasia. Their incidence depends on the treated area and the regimen of radiotherapy administered; hyperfractionated schedules are usually associated with a lower occurrence of late side effects and vice versa [30, 31].

### 2.4. Cryosurgery

Cryosurgery is based on the application of liquid nitrogen at −196.5°C to destroy tumor cells through the direct effect of freezing and vascular stasis.

Tissue damage depends on intracellular and extracellular crystals formation.

For cSCC, a rapid cooling is preferred since it leads to faster intracellular crystal formation that results in better destruction of tumor cells.

After treatment, patients may exhibit vesiculation, erythema, exudation, and edema, but after a 4- to 6-week period the damaged area usually heals without sequelae.

Hypopigmentation is the main side effect of cryosurgery due to melanocytes destruction during freezing.

This method is recommended for treating cSCCs with well-defined borders in elderly and disable patients.

### 2.5. Curettage and Electrodesiccation

Curettage and electrodesiccation is a destructive technique often used to treat superficial low risk cSCCs on the trunk and extremities, namely, very differentiated forms. It is preferred in elderly people. After a local anesthesia, the friable tumor tissue is scraped away by curettage and then the area is electrodesiccated to cause necrosis of residual cells.

For superficial lesions, one cycle may suffice. The area then heals by second intention, which usually results in a pink to white roundish scar.

Similarly to cryosurgery, this approach does not permit histologic examination and it is not recommended for high risk tumors, lesions larger than 2 cm in diameter, or recurrent tumors [32].

### 2.6. Chemotherapy

#### 2.6.1. Oral Chemotherapy

**Figure 5:**
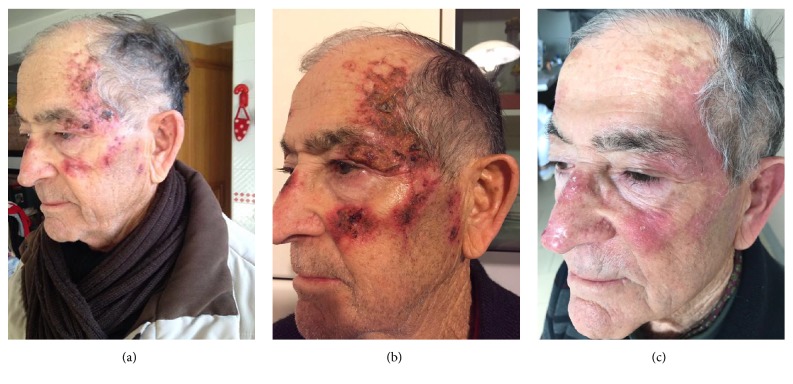
Before (a), during (b), and after (c) treatment with 5-FU of hyperkeratotic extensive squamous cell carcinoma.

Capecitabine is an oral prodrug of 5-FU that may be a valuable substitute of infusional 5- FU [33, 34]. Cartei et al. [35] prospectively investigated the efficacy of oral capecitabine in 14 patients with SCC that had not been eradicated by surgery, radiotherapy, and topical 5-FU (Figures 5(a), 5(b), and 5(c)).

The low-dose of capecitabine administrated resulted in appreciable improvement in 5 patients and arrested tumor growth in 4 patients.

#### 2.6.2. Intravenous Chemotherapy

Intravenous chemotherapy may be used for SCC in patients with distant metastasis, when surgery and radiotherapy failed or when these treatment are contraindicated.

Platinum compounds represent the standard choice; besides they have been combined with paclitaxel [36], 5-FU [37], and adriamycin [38].

In 1999 Denic et al. combined platinum compounds with bleomycin in patients with inoperable SCCs. This combination resulted in improved tumor resectability in 2 out of 3 patients, including one patient with xeroderma pigmentosum [39].

A recent comparative study reported cisplatin as a promising agent for the treatment of local invasive cutaneous squamous cell carcinoma with respect to the 5-fluorouracil. An optimal cisplatin-based chemotherapy might provide a better outcome in patients with an invasive cSCC rather than surgery [40].

Also combined use of chemotherapy and local therapy (surgery and/or radiotherapy) has been described [41–43]; in particular, chemotherapy has been used in a neoadjuvant setting.

Furthermore, palliative chemotherapy combined with radiotherapy in patients affected by mucosal SCCs of head and neck showed higher survival rates compared to radiotherapy alone [44, 45].

A multicenter study involving patients with advanced primary, recurrent, or metastatic skin tumors of the extremities, including 12 SCCs, showed that hyperthermic isolated limb perfusion with tumor necrosis factor alpha (TNF-alpha), interferon gamma (IFN-gamma), and melphalan improved the locoregional control of the disease, saving the majority of patients from limb amputation [46].

### 2.7. Electrochemotherapy

This procedure involving electroporation combined with antineoplastic drug can represent a new conservative option for the treatment of extensive cSCC in which surgical procedures would have entailed wide tissue sacrifice. A retrospective single-center study enrolling 22 patients showed responses in 18 (81.8%) patients, assessing the safety and effectiveness of this procedures [33].

### 2.8. Biological Response Modifiers (BRMs)

Biological response modifiers (BRMs) have been used in oncology to increase host antitumor immune activity. In SCC, there is lack of data about the use of BRMs for advanced stages.

Because in vitro studies demonstrated synergism between retinoids and interferons [47], these agents have been used in combination. In particular, two phase II studies employing a combination of interferon alpha-2a and 13-cis-retinoid (13-cRA), with or without cisplatin, showed some clinical activity in extensive locally advanced tumors [34, 37].

In literature, the concurrent use of BRMs and chemotherapy has been investigated [48]. Shin et al. [49] conducted a phase II trial combining interferon alfa and cisplatin with 13-cRA in patients with locally advanced SCC; the 67% of patients showed an effective clinical improvement with a median duration of 35 months.

Some patients referred mucocutaneous dryness, mild to moderate fatigue, and moderate to severe neutropenia during BMRs treatment.

### 2.9. Targeted Therapy

Epidermal growth factor receptor (EGFR) is commonly expressed in cutaneous SCC of the face and trunk, as well as in lymph node metastases; further, EGFR overexpression has been associated with a worse prognostic outcome [50].

Cetuximab is a chimeric human and murine anti-EGFR monoclonal antibody, currently approved for the treatment of metastatic head and neck SCCs; on the contrary its use as second line treatments after mono- or polychemotherapy failure is discussed.

In the EGFR inhibitors group there are also small molecule kinase inhibitors (erlotinib, gefitinib, and dasatinib) that have been approved for the treatment of head and neck SCC.

In a phase II study on 23 patients with locally aggressive SCC, the use of gefitinib for 2 cycles as a neoadjuvant treatment followed by surgery and/or radiotherapy showed an overall response rate of 45.5% with a 2-year disease specific survival rate of 72% and a progression-free survival rate of 63% [51]. Moreover, another phase II study on 36 patients with unresectable SCC treated with cetuximab reported a response rate of 25% and a disease stabilization in 42% of cases [52]. On the other hand, a randomized phase III study on 117 patients with metastatic head and neck SCCs revealed that cetuximab combined with a standard regimen of cisplatin improved response rates but did not have any a significant effect on overall and progression-free survival [53].

In conclusion, EGFR inhibitor may constitute an interesting therapeutic option, but literature data are still insufficient and this approach is currently under evaluation.

### 2.10. New Compounds under Study

#### 2.10.1. Herbacetin

Herbacetin is a flavonol compound that is found in plants; it possesses a strong antioxidant capacity and exerts anticancer effects on colon and breast cancer. Recently in vivo and in vitro studies on cSCC and melanoma cell growth have been carried out identifying herbacetin as a dual V-akt murine thymoma viral oncogene homolog (AKT) and ornithine decarboxylase (ODC) inhibitor.

Results of cell-based assays showed that herbacetin inhibits neoplastic transformation of cutaneous SCC and melanoma cells. These preliminary results need further clinical investigations [54].

#### 2.10.2. Wool Hydrolysates

A recent study highlights the bioactive properties of wool hydrolysates on cSCC cells, decreasing their number. The authors of the study hypothesize that wool hydrolysates may be promising agents to be used topically for treatment of transformed keratinocytes in actinic keratosis and invasive squamous skin cancer in humans [55].

#### 2.10.3. Immunotherapy: Future Perspectives and Ongoing Trials

The role of immunotherapy in the treatment of squamous cell carcinoma of the skin is under investigation. The ASCO Post from the ASCO Meeting 2017 reported the first evidence that PD-1 inhibitors may have a role in the management of advanced cSCC [56]. This was a very early report, whose promising results need to be confirmed by larger scale studies. REGN2810, a fully human monoclonal antibody targeting PD-1, was well tolerated in patients with advanced cSCC. A pivotal trial of REGN2810 for patients with advanced CSCC is ongoing (NCT02760498), so the results from this trial will further elucidate these previous interesting results.

## 3. Discussion

cSCC is the second most common skin cancer worldwide. A well-established relationship exists between cSCC and ultraviolet (UV) radiation, especially UVB. Arsenic exposure and the human papilloma virus (types 6, 11, and 16) are other risk factors associated. Among NMSC, cSCC has a greater propensity for invasive behaviour and metastasis. cSCC involving the scalp, forehead, ears, nose, and lips has the highest risk of metastasis such as undifferentiated lesions greater than 6 mm thick that have proceeded to invade deeper structures, including the musculature, perichondrium, or periosteum [57].

Surgery is the primary means of treatment for squamous cell carcinoma of the skin.

Mohs' micrographic surgery is the treatment of choice for squamous cell carcinoma of the head and neck, in immunosuppressed patients, recurrent squamous cell carcinoma, squamous cell carcinoma with aggressive histologic features, and squamous cell carcinoma greater than or equal to 2 mm of depth. The American Academy of Dermatology guidelines help select those cases that would most benefit from the Mohs procedure, while conserving healthcare expenditures [58]. For in situ disease, electrodesiccation with curettage, or topical treatment with 5FU, imiquimod, and photodynamic therapy have been used successfully [59].

Radiation therapy is likely most beneficial in the adjuvant setting for high risk cSCC on head neck and mucosa. Chemotherapy is typically best reserved for patients with metastatic or locally advanced disease that is not controllable with surgical and/or radiation therapies. EGFR inhibitors and immunotherapies are newer targeted treatments and may offer greater efficacy in these settings [60].

Patients with a history of a few squamous cell carcinomas and some actinic keratoses may be followed every six to 12 months, while those with many squamous cell carcinomas or aggressive tumors likely will need to be seen much more often [59].

Selecting the most appropriate therapeutic strategy, the clinical and histological features of the lesion, the patient aspect and the body area involved should be considered. Nonsurgical therapies can be used in elderly patients with comorbidity and nonaggressive tumors. Lesions arising on mucosa and nearby sense organs benefit from more aggressive treatments and closer follow-up. The surgical treatments of the head-neck district require high surgical skills in order to guarantee radicality of the surgical excision and conservation of the aesthetic-functional units of the treated area. In the case of demolition therapies such as electrodesiccation and laser therapy, it is recommended to previously perform biopsy with histological verification in order to limit legal medical controversies.

## 4. Conclusions

Skin carcinogenesis is a multistep process with several stages along its malignant evolution.

cSCC can be invasive or not, requiring these forms different approaches. To date, surgery is still considered as the gold standard approach for invasive cSCC therapy. Metastasis to regional lymph nodes occurs in approximately 5% of cases and treatment involves a combination of surgery and adjuvant radiation. Nevertheless, in recent years new nonsurgical modalities have shown high efficacy rates and could be considered in selected cases, such as elderly and/or disable patients when surgery is contraindicated [61]. New insights from Genome-wide association studies (GWAS) identifying genetic loci associated with cutaneous squamous cell carcinoma (cSCC) risk and invasiveness may help identify individuals at higher risk for developing clinically aggressive cSCC. These interventions will guide the sparing of surgical intervention. Moreover novel agents, such as herbacetin and wool hydrolysates, are currently under investigation. miRNA represents a potential biomarker along the malignant evolution of keratinocytes towards cSCC and further studies on this interesting and promising field are warranted [62, 63].
